# Supplementary material for: Altered food liking in depression is driven by macronutrient composition
Source: Psychol Med. 2025 Feb 5;55:e20. doi: 10.1017/S0033291724003581 (PMC12017361; doi:10.1017/S0033291724003581)
Supplement: Thurn et al. supplementary material [file S0033291724003581sup001.docx]

**Supporting Information**

# Altered food liking in depression is driven by macronutrient composition

Lilly Thurn^1^, Corinna Schulz^1,2^, Diba Borgmann^4^, Ebru Sarmisak^2^, Johannes Klaus^2^, Sabine Ellinger^5^, Martin Walter^2,6-8^, & Nils B. Kroemer^1-3*^

^1^ Section of Medical Psychology, Department of Psychiatry & Psychotherapy, Faculty of Medicine, University of Bonn, Bonn, Germany

^2^ Department of Psychiatry & Psychotherapy, Tübingen Center for Mental Health, University of Tübingen, Tübingen, Germany

^3^ German Center for Mental Health (DZPG), partner site Tübingen

^4^ Novo Nordisk Foundation Center for Basic Metabolic Research, Faculty of Health and Medical Sciences, University of Copenhagen, Denmark

^5^ Institute of Nutritional and Food Sciences, Human Nutrition, University of Bonn, Bonn, Germany

^6^ Department of Psychiatry & Psychotherapy, University Hospital Jena, Jena, Germany

^7^ Department of Behavioral Neurology, Leibniz Institute for Neurobiology, Magdeburg, Germany

^8^ German Center for Mental Health (DZPG), partner site Jena-Magdeburg-Halle

**Corresponding author***

Prof. Dr. Nils B. Kroemer, [nkroemer@uni-bonn.de](mailto:nkroemer@uni-bonn.de)

Venusberg Campus 1, 53127 Bonn, Germany

**SI1. Antidepressant medication**

| **Characteristic** | **Overall**  N = 117 | **HCP**  N = 63 | **MDD**  N = 54 |
| --- | --- | --- | --- |
| **Antidepressive (AD) medication, n (%)** |  |  |  |
| no_AD | 92 (79) | 63 (100) | 29 (54) |
| SSRI | 13 (11) | 0 (0) | 13 (24)^1^ |
| Other | 12 (10) | 0 (0) | 12 (22)^2^ |
| *^1^* Escitalopram (6x), Sertralin (7x) *^2^* Venlafaxin (5x), Duloxetin (2x), Bupropion (2x), Agomelatin (1x), Mirtazapin (1x), Pipamperon (1x) | | | |

**SI2. Hormone measurements**

“Monovettes were transferred to the Central Laboratory of the Institute of Clinical Chemistry and Pathobiochemistry of the University Hospital Tübingen for analysis of glucose, insulin, and triglycerides in plasma or serum. Glucose was determined in sodium fluoride plasma using an enzymatic test kit (Atellica CH Glucose Hexokinase_3; Atellica Solution, CI analyser), insulin in serum using an immunological assay (Atellica IM Insulin; Atellica Solution, IM Analyzer) and triglycerides in lithium heparin plasma using of an enzymatic assay (Atellica CH Triglycerides_2, Atellica Solution, CI Analyzer; Siemens Healthineers, Eschborn, Germany; within-laboratory precision for glucose ≤ 2.2%, for insulin ≤ 10%, and for triglycerides ≤ 4.0% according to manufacturer). Plasma samples for analysis of ghrelin were obtained from K3E-EDTA Monovettes immediately by centrifugation of the blood samples at 4°C with 2000 g for 10 min. Then, 500 µl of plasma was transferred into two cooled cryo tubes (Thermo Scientific™ Nunc™) each and 50 µl of cooled 1 M hydrochloric acid (HCl) in plasma to acid ratio of 10:1 was added to each tube to prevent ghrelin from deacetylating. The tubes were immediately capped, gently reversed, and cooled at -20°C before they were stored at -80°C (after 24 to 48 h). After completing the trial, the frozen samples were transferred on dry ice to the University of Bonn. The concentration of both acylated and unacetylated ghrelin was determined by using ELISA kits (#A05306 and #A05319; both from Bertin Bioreagent, Bertin Technologies, Montigny-le-Bretonneux, France; distributed by BioCat, Germany) at the Institute of Nutritional and Food Sciences, Human Nutrition.” (Fahed et al., 2024; Schulz et al., 2023)

**SI3. Macronutrient composition of the selected foods**

| **Description** | **Energy_kcal_100g** | **TotalFat_g_100g** | **SaturatedFat_g_100g** | **Carbohydrate_g_100g** | **TotalSugar_g_100g** | **Protein_g_100g** | **Fiber_g_100g** |
| --- | --- | --- | --- | --- | --- | --- | --- |
| Milk chocolate | 546.0 | 32.5 | 20.7 | 54.9 | 49.0 | 6.9 | 2.8 |
| Biscuit with chocolate | 469.0 | 20.0 | 10.7 | 62.5 | 30.1 | 7.2 | 4.7 |
| Mars | 447.0 | 16.6 | 7.7 | 70.1 | 64.0 | 3.8 | 1.2 |
| Chocolate Cupcake iced with nuts | 548.4 | 20.0 | 10.1 | 58.5 | 38.0 | 7.0 | 0.9 |
| Bonbons (chocolates) | 519.0 | 30.8 | 16.8 | 53.1 | 51.8 | 5.6 | 3.3 |
| Whipped cream pie | 350.0 | 25.0 | 11.6 | 29.0 | 23.1 | 3.5 | 0.8 |
| Mini donuts | 358.0 | 21.1 | 4.2 | 35.5 | 4.8 | 6.0 | 1.1 |
| Kinder Bueno/Children's Bueno | 571.0 | 37.3 | 17.3 | 49.5 | 41.3 | 9.2 | 2.0 |
| Smarties 1 | 465.0 | 17.9 | 9.7 | 72.1 | 65.4 | 3.9 | 2.7 |
| Chocolate bar | 549.6 | 32.6 | 20.3 | 56.8 | 54.4 | 6.7 | 1.1 |
| Kitkat | 520.0 | 27.7 | 15.2 | 59.0 | 48.6 | 7.1 | 3.0 |
| Cake with chocolate | 450.0 | 25.0 | 14.8 | 50.0 | 32.4 | 5.5 | 2.3 |
| Chocolate chip cookies | 500.0 | 25.0 | 13.0 | 60.8 | 31.8 | 6.4 | 2.9 |
| Strawberry candies | 360.0 | 0.0 | 0.0 | 90.0 | 65.0 | 0.1 | 0.7 |
| Raisins | 326.0 | 0.5 | 0.2 | 75.5 | 72.8 | 3.1 | 3.7 |
| Strawberries with whipped cream | 75.6 | 4.4 | 2.9 | 6.9 | 6.7 | 0.9 | 0.9 |
| Pancakes | 196.0 | 4.9 | 2.1 | 29.2 | 4.9 | 8.3 | 0.6 |
| Strawberry pie with whipped cream | 205.0 | 11.0 | 4.7 | 24.0 | 13.0 | 2.5 | 1.7 |
| Apple (green) | 60.0 | 0.2 | 0.0 | 13.0 | 10.3 | 0.2 | 2.0 |
| Fruitsalad (berries, banana, melon) | 56.4 | 0.1 | 0.0 | 10.8 | 8.7 | 0.7 | 3.2 |
| Grapes, white | 78.0 | 0.2 | 0.1 | 16.8 | 16.2 | 0.5 | 1.7 |
| Tangerine | 45.0 | 0.1 | 0.0 | 9.8 | 8.2 | 0.7 | 0.9 |
| Kiwi, halves | 68.0 | 0.8 | 0.2 | 12.2 | 10.3 | 0.9 | 2.3 |
| Fruit salad | 45.7 | 0.2 | 0.0 | 9.5 | 8.7 | 0.4 | 1.3 |
| Nectarine | 33.0 | 0.1 | 0.0 | 6.5 | 6.5 | 1.0 | 1.1 |
| Orange | 51.0 | 1.0 | 0.2 | 7.8 | 7.7 | 0.8 | 2.0 |
| Cherries | 57.0 | 0.0 | 0.0 | 13.0 | 13.0 | 0.0 | 1.2 |
| Watermelon | 38.0 | 0.0 | 0.0 | 8.0 | 8.0 | 1.0 | 0.6 |
| Red berries | 48.0 | 0.0 | 0.0 | 5.0 | 4.0 | 1.0 | 8.2 |
| Black berries | 37.0 | 0.0 | 0.0 | 5.1 | 5.1 | 0.9 | 3.1 |
| Potato crisps (natural) | 541.0 | 33.5 | 5.9 | 51.3 | 0.5 | 6.4 | 4.1 |
| Cheese twist | 508.0 | 32.2 | 17.2 | 41.0 | 2.7 | 12.8 | 1.5 |
| Nuts | 577.0 | 47.0 | 6.9 | 11.0 | 3.5 | 24.0 | 7.0 |
| Crackers with medium mature white cheddar cheese | 415.0 | 28.9 | 15.6 | 17.0 | 2.0 | 21.2 | 0.6 |
| Nacho-cheese tortilla chips | 487.0 | 22.3 | 9.4 | 62.5 | 1.3 | 6.9 | 4.3 |
| Pepper potato crisps | 544.0 | 33.0 | 5.8 | 53.5 | 2.5 | 6.3 | 4.0 |
| Cheese plate | 338.0 | 28.0 | 20.3 | 0.3 | 0.3 | 21.2 | 0.0 |
| French fries with sauce | 367.7 | 24.1 | 15.5 | 32.4 | 1.0 | 3.9 | 2.7 |
| Cream cheese on toast | 370.3 | 9.9 | 8.1 | 53.1 | 2.5 | 16.1 | 2.7 |
| Spanish sausage | 358.0 | 29.1 | 11.3 | 3.4 | 3.0 | 20.7 | 0.0 |
| Cream crackers | 465.0 | 17.0 | 7.0 | 70.0 | 5.0 | 8.0 | 2.2 |
| Pretzels | 395.0 | 7.3 | 1.2 | 70.0 | 3.3 | 12.0 | 4.2 |
| Cocktail nuts | 547.0 | 33.2 | 7.6 | 44.3 | 4.6 | 15.4 | 4.8 |
| Bake rolls | 447.0 | 15.0 | 7.0 | 62.0 | 5.0 | 14.0 | 4.0 |
| Mini Snack a Jacks | 414.0 | 2.2 | 0.8 | 73.0 | 2.7 | 6.7 | 1.1 |
| Pizza bolognese slice | 220.0 | 9.0 | 3.1 | 25.0 | 2.0 | 9.0 | 2.0 |
| Pepper, yellow | 23.0 | 0.2 | 0.0 | 3.5 | 3.4 | 0.7 | 2.5 |
| Radish | 22.0 | 0.0 | 0.0 | 4.0 | 3.0 | 1.0 | 0.9 |
| Eggs, halves | 136.0 | 8.8 | 2.9 | 1.9 | 0.0 | 12.3 | 0.0 |
| Tomato, cut | 23.0 | 0.5 | 0.1 | 3.1 | 3.0 | 0.7 | 1.4 |
| Sweet pepper, red | 28.0 | 0.1 | 0.0 | 5.0 | 4.4 | 0.8 | 1.8 |
| Carrot | 33.0 | 0.3 | 0.1 | 5.5 | 4.3 | 0.6 | 2.8 |
| Cucumber | 13.0 | 0.2 | 0.1 | 1.9 | 1.7 | 0.6 | 0.6 |
| Sweet corn | 74.0 | 1.4 | 0.4 | 11.6 | 8.1 | 2.5 | 2.5 |
| Sushi | 165.0 | 3.5 | 0.1 | 28.0 | 6.3 | 6.0 | 0.8 |
| Greek salad | 92.0 | 7.2 | 3.1 | 2.2 | 0.6 | 4.0 | 1.1 |
| Mini peppers | 22.0 | 0.1 | 0.0 | 3.4 | 3.1 | 0.8 | 2.3 |
| Olive, pepper and cucumber salad | 57.9 | 3.8 | 2.8 | 1.7 | 2.0 | 3.6 | 1.2 |
| Mini pickles (sour) | 10.0 | 0.0 | 0.0 | 1.0 | 1.0 | 1.0 | 0.9 |
| Olives | 111.0 | 11.0 | 1.6 | 0.5 | 0.0 | 0.9 | 4.0 |

**SI4. Metabolic state ratings before the FCR.**

**
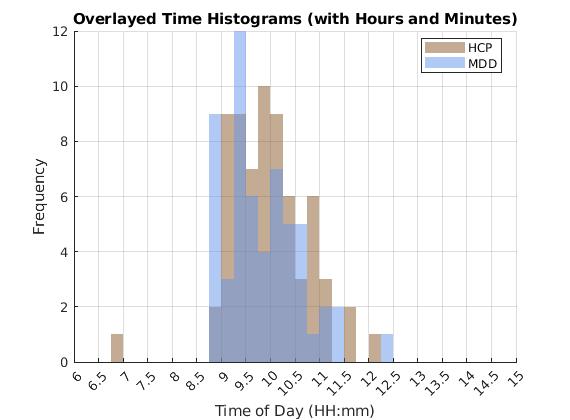

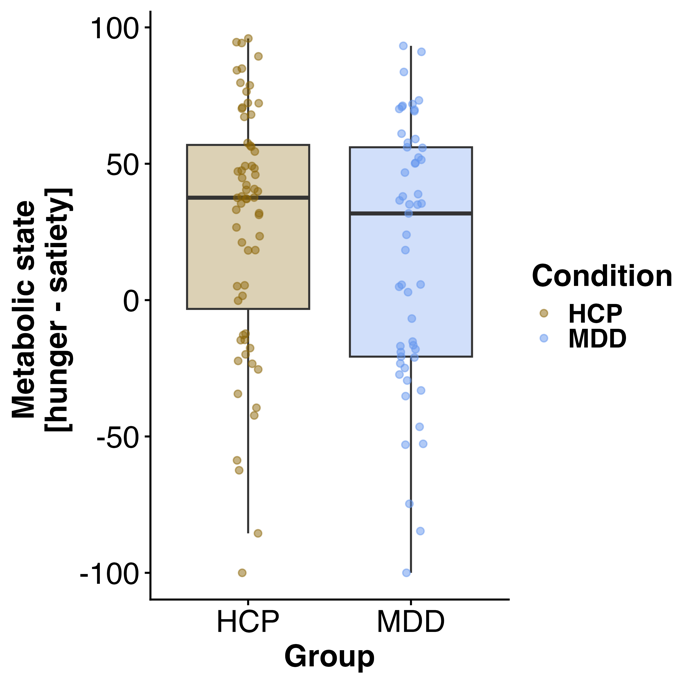
**

**Fig S4.** Patients and healthy controls do not differ in their metabolic state ratings at the beginning of the session (left, *b* = -10.75, *p* = 0.22), i.e., time of the blood draw roughly 20 min before the FCR and started the metabolic state assessment roughly at the same time (right; *t* = 0.97, *p* = 0.34).

**SI5. Wanting and Liking Ratings for individual food items**


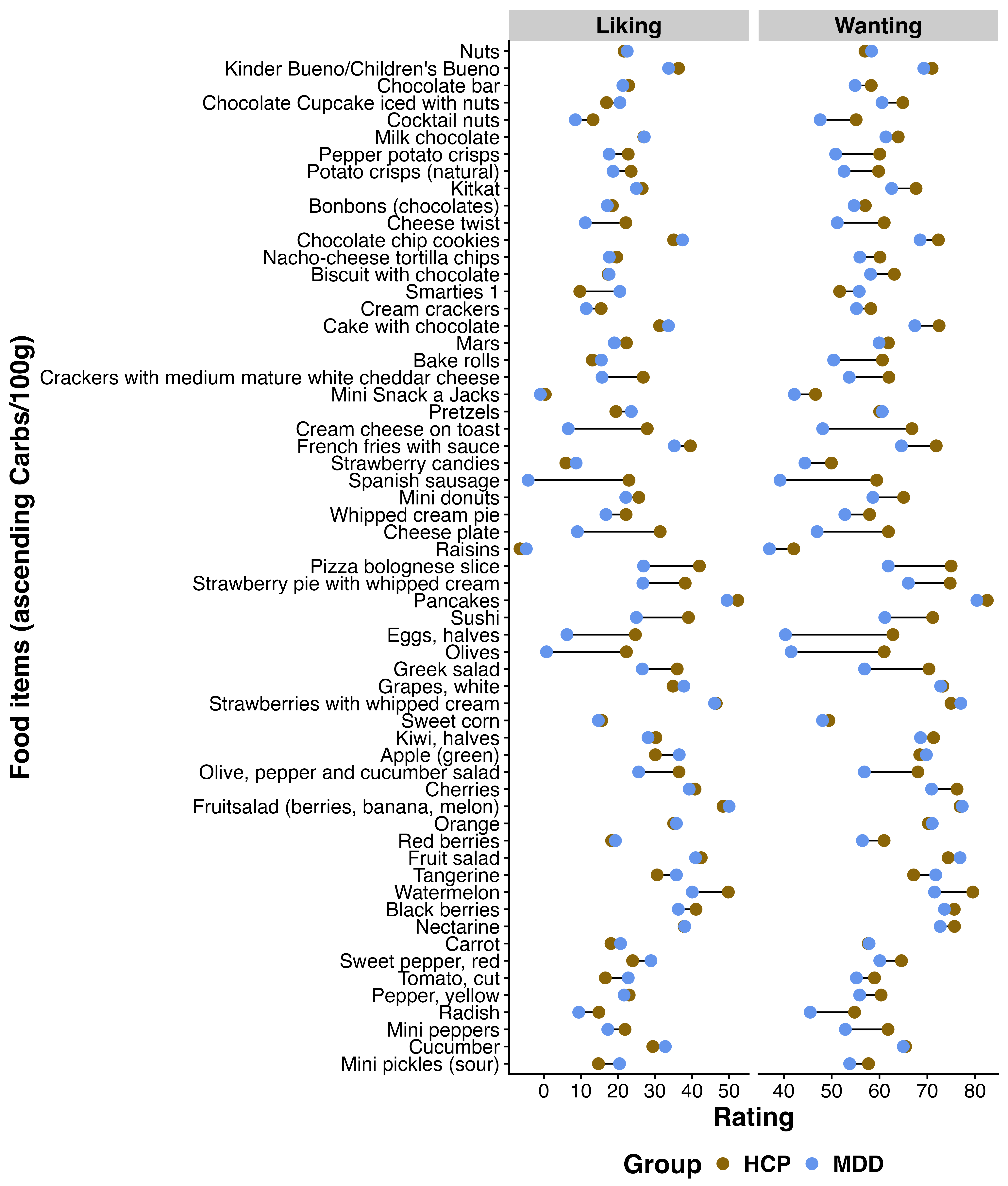


**Fig S5.** Liking and Wanting ratings (untransformed) for individual food items, sorted in ascending order for carbs/100g per group (HCP vs. MDD).

**SI6. Protein and Fat**


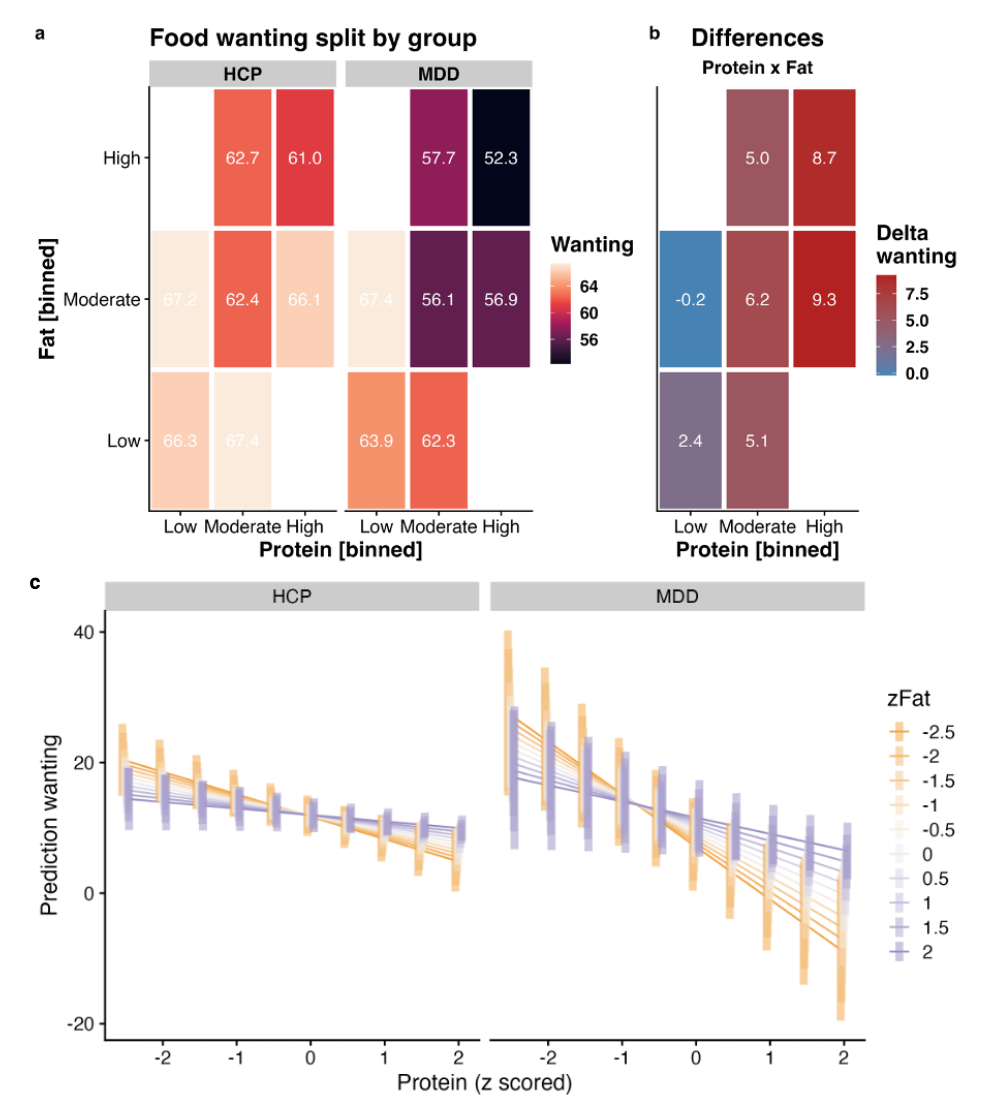


**Fig. S6:** Patients with major depressive disorder (MDD) report lower wanting for foods with high protein unless they contain fat as well. A: Wanting ratings split by group and the macronutrients protein and fat. For display, we binned content so that low corresponds to the lower quartile, moderate to the second and third quartile, and high to the upper quartile. There is no data for food items high in protein and low in fat as well as low in protein and high in fat since the set of food items was not balanced in this regard. B: Differences between healthy control participants (HCP) and patients with MDD for foods according to their protein and fat content. Patients with MDD report lower wanting for high-protein foods (interaction contrast: *p* = .004), unless they are high in fat (p = .019). C: Visualization of the model prediction that patients with MDD gave higher ratings for wanting (*p* = .019) if foods were rich in both protein and fat.

**SI7. Macronutrient preference and anxiety**


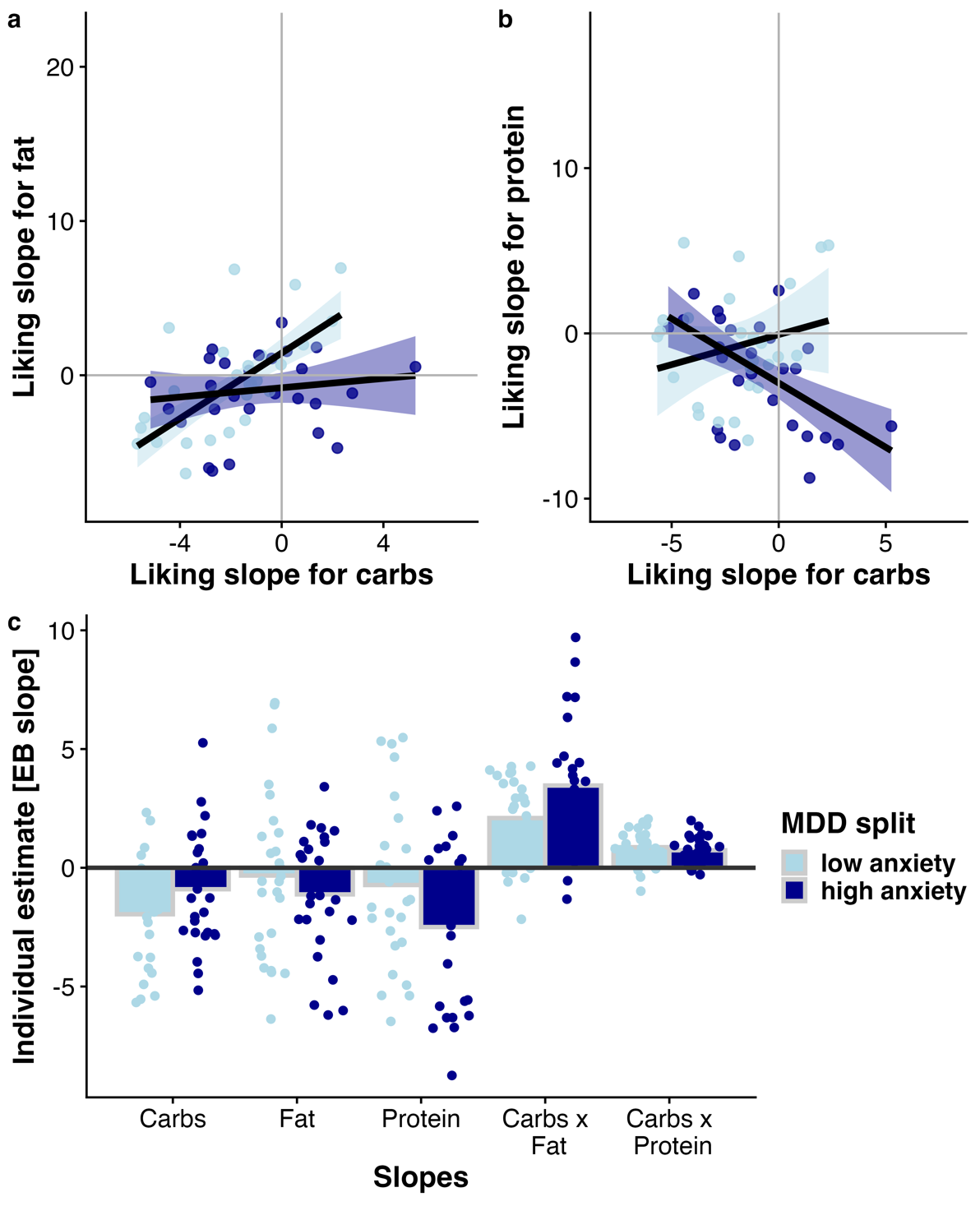


**Fig. S7.** Patients with high anxiety show attenuated associations between liking slopes for carbohydrates and fat as well as protein. Patients with depression were split according to the STAI-T (median split) into a low and high anxiety for descriptive purposes. A: Patients with high anxiety show a weaker correlation between individual estimates of increases in liking per one unit of carbohydrate content (i.e., liking slopes) and increases in liking for fat. B: Patients with high anxiety show a negative correlation between liking slopes for carbohydrates and protein. C: Individual estimates per macronutrient split by anxiety. The unbiased empirical Bayes (EB) estimates were derived from linear mixed-effects models that did not include anxiety as a regressor. Patients with high anxiety vs. low anxiety show a similar pattern of estimates as MDD vs. HCP (Figure 4).

**SI8. Hormonal values**

| **Characteristic** | **Overall**  N = 117 | **HCP**  N = 63 | **MDD**  N = 54 | **Test Statistic**^2^ | **p-value**^2^ |
| --- | --- | --- | --- | --- | --- |
| **Acyl ghrelin**^1^ **[pg/mL]), Mean (±SD)** | 173.8 (±206.5) | 182.2 (±204.6) | 166.2 (±210.0) | 0.38 | .71 |
| Missing | 20 | 17 | 3 |  |  |
| **Des-acyl ghrelin**^1^ **[pg/mL], Mean (±SD)** | 188.2 (±103.6) | 186.5 (±94.1) | 189.8 (±112.4) | -0.15 | .88 |
| Missing | 20 | 17 | 3 |  |  |
| **Glucose [mg/dl], Mean (±SD)** | 83.9 (±7.7) | 83.1 (±6.9) | 84.8 (±8.5) | -1.10 | .26 |
| **Insulin [mg/dl], Mean (±SD)** | 60.1 (±36.7) | 52.6 (±27.0) | 68.9 (±44.2) | -2.40 | .020 |
| **HOMA IR, Mean (±SD)** | 1.8 (±1.2) | 1.6 (±0.9) | 2.1 (±1.5) | -2.40 | .020 |
| **Triglyceride Index, Mean (±SD)** | 4.4 (±0.3) | 4.4 (±0.3) | 4.5 (±0.3) | -1.20 | .23 |

^1^ Values of acyl and des-acyl ghrelin refer to data of 97 participants. ^2^ Pearson’s Chi-squared test; Welch Two Sample t-test.

Data are means ± SD if not indicated otherwise*. Abbreviations*: HCP = healthy control participants, MDD = major depressive disorder, HOMA-IR = homeostasis model assessment of insulin resistance, TyG = Triglyceride-glucose Index, BDI = Beck’s Depression Inventory, SHAPS = Snaith-Hamilton Pleasure Scale, STAI-T = State Trait Anxiety Inventory – Trait.

**SI9. Ghrelin and liking ratings**

Both higher fasting levels of acyl (*r* = .257, *p* = .011) and desacyl ghrelin (*r* = .251, *p* = .013) were associated with higher overall liking ratings, with similar correlation coefficients in both groups for acyl (r_HCP_ = .25, r_MDD_ = .23) and des-acyl ghrelin (r_HCP_ = .22, r_MDD_ = .30)**.**
